# Closing the Loop on Phosphorus Loss from Intensive Agricultural Soil: A Microbial Immobilization Solution?

**DOI:** 10.3389/fmicb.2018.00104

**Published:** 2018-02-06

**Authors:** Lin Zhang, Xiaodong Ding, Yi Peng, Timothy S. George, Gu Feng

**Affiliations:** ^1^Department of Ecological Science and Technology, College of Resources and Environmental Sciences, China Agricultural University, Beijing, China; ^2^Department of Soil Science and Plant Nutrition, College of Resources and Environment, Qingdao Agricultural University, Qingdao, China; ^3^James Hutton Institute, Dundee, United Kingdom

**Keywords:** agricultural soil, carbon: phosphorus ratio, increase carbon input, microbial biomass phosphorus, phosphorus pollution

## Introduction

Phosphorus (P) fertilizer has been applied profligately across the globe, being particularly overused in China in the past 30 years in order to pursue high yields (Cordell et al., 2009; Li et al., 2014). This has greatly increased the P content of soil in various intensive agricultural systems (including cereals, vegetables, and fruit orchard), many of which now contain sufficient P to potentially supply P for adequate yields in these crops for several years (Li et al., 2011; Tóth et al., 2014). For example, the soil available P in some cereal crop, vegetable and orchard systems in China have arrived at 24.7, 181, and 43.1 mg kg^−1^, respectively (Lu, 2009; Li et al., 2011; Kalkhajeh et al., 2017). Over-application of P fertilizer is in itself wasteful, but the transport of excessive P from soil solution to the waterbodies by surface runoff and leaching causes various environmental problems, including eutrophication of lakes, rivers and near coastal zones, pollution of ground water aquifers, algal blooms, and the loss of terrestrial and aquatic biodiversity (Chen et al., 2008; Schoumans et al., 2014; Smith et al., 2015). Consequently, it is imperative to understand how best to immobilize P in the soil to avoid its loss to the wider environment. This is an urgent environmental issue that should be considered as a priority in intensive agricultural systems across the globe, but particularly in China.

## Phosphorus immobilization by soil microbes

Soil microbes including bacteria, fungi, and microfauna play important roles in the biogeochemical cycle of P and are involved in both mineralization and immobilization of P (Richardson and Simpson, 2011). On the one hand, soil microbes are capable of mobilizing organic P and non-soluble inorganic P (Jorquera et al., 2008) by exuding protons, carboxylates and phosphatases to release the orthophosphate, usually H_2_${\text{PO}}_{4}^{-}$ and ${\text{HPO}}_{4}^{2-}$, for their own and root uptake (Rodríguez and Fraga, 1999). The phosphate solubilizing bacteria usually belong to the genera of *Pseudomonas, Bacillus, Rhizobium, Burkholderia, Achromobacter, Agrobacterium, Microccocus, Aereobacter, Flavobacterium*, and *Erwinia* (Rodríguez and Fraga, 1999). The phosphate solubilizing fungi usually belong to the genera of *Aspergillus, Trichoderma*, and *Penicillium* (Whitelaw, 1999). On the other hand, soil microbes can also transform available P into microbial biomass P (MBP) to allow the use of organic carbon (C) and root exudates for energy (Wu et al., 2007). The immobilized P can be released to increase available P during the microbial biomass turnover. The turnover time of MBP in the field can range from tens of days to near one year (Chen and He, 2002; Kouno et al., 2002; Liebisch et al., 2014), which is largely dependent on a range of environmental factors, e.g., soil moisture, season, and application of fertilizers (Patra et al., 1990; He et al., 1997; Butterly et al., 2009). Liebisch et al. (2014) estimate the flux of P through the microbial biomass can arrive at 18.1–36.9 kg P ha^−1^ season^−1^ (from March to November). Most studies usually focus on how to use soil microbes to mobilize soil P (Richardson and Simpson, 2011), neglecting how to exert their functions on P immobilization.

**Figure 1:**
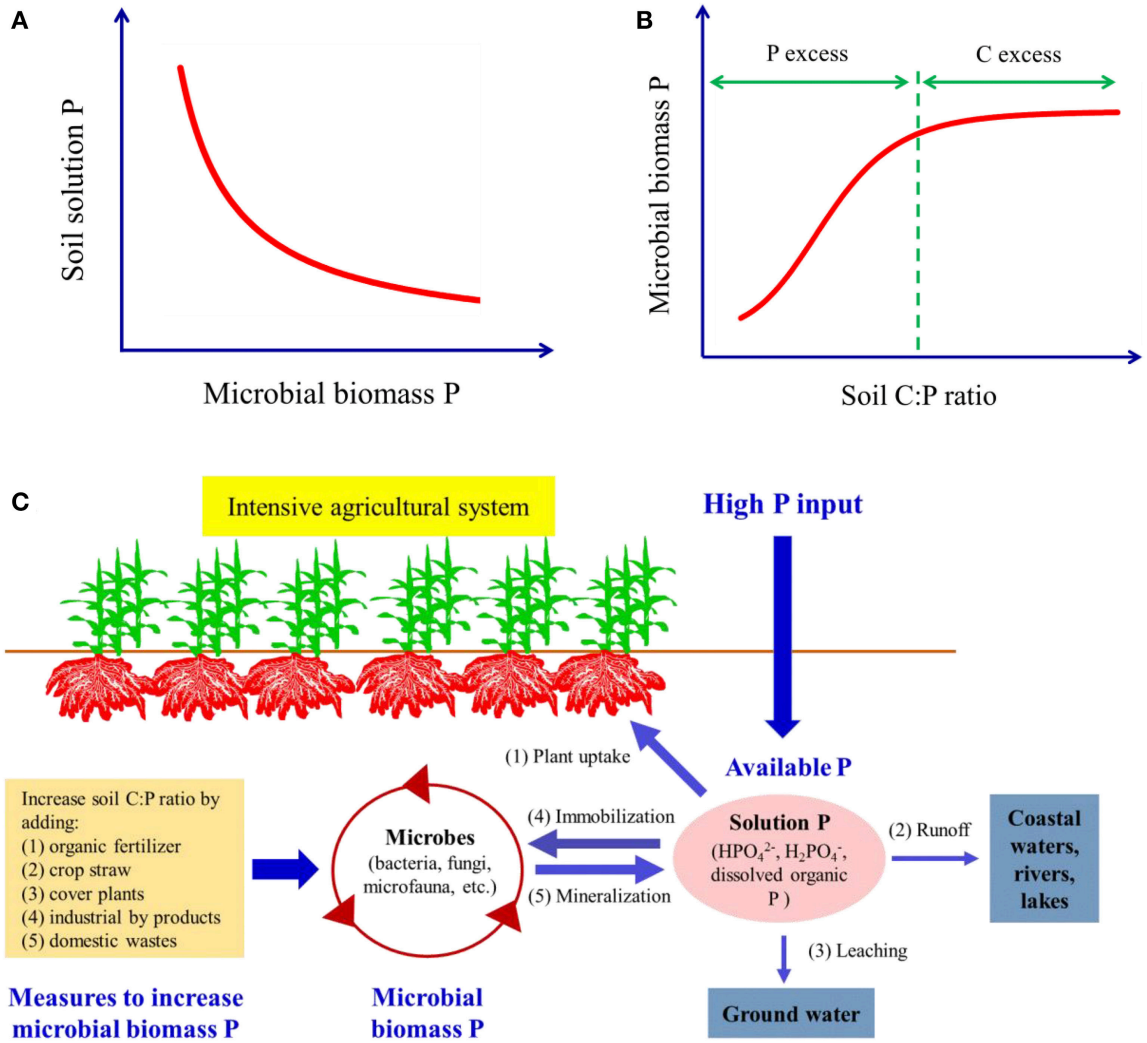
**(A)** The relationship between microbial biomass P and soil solution P; **(B)** the relationship between soil C:P ratio and microbial biomass P; **(C)** the measures to increase microbial biomass P by increasing soil C:P ratio to reduce P lost.

Indeed, MBP is negatively related to soil solution P (Figure 1A) and has strong competition with soil available P (Kouno et al., 2002). However, approaches to manipulate soil microbes to immobilize more P to reduce P loss have been paid little attention. Among various abiotic and biotic factors that can influence the dynamics of MBP, soil carbon (C):P ratio is an important one (Marschner, 2008). However, soil P pools are complex and different P pools, e.g. total P, available P and organic P can be used to calculate the C:P (Stevenson, 1986; Cleveland and Liptzin, 2007; Spohn and Widdig, 2017). Which of these reflects a better relationship with MBP is still unclear and needs further investigation. Generally, at low C:P ratio, MBP is small because of the C limitation (Cleveland and Liptzin, 2007). As the C:P ratio increases, MBP will increase through further microbial immobilization for the input of C (Kouno et al., 2002). At high C:P ratio, MBP becomes stable because C is in excess and P is relatively limited (Figure 1B). Evidence suggest that soil C:P ratio in the Chinese intensive agricultural system is less than the equilibrium and P is normally the element in excess compared with C (Tian et al., 2010). Moreover, due to excessive application of P fertilizer, the C:P ratio has become even lower in recent years (Xu et al., 2015). This suggests there is an opportunity to raise the C:P ratio of Chinese soils and lock up some of this excess P by forcing microbial immobilization.

## Microbial immobilization of phosphorus is limited by C:P ratio

The comparison of the present and initial value of C:P ratio in the P addition treatment of 22 long-term field experiments shows that the C:P ratio decreased, on average, by 13% over the course of the experiments (Xu et al., 2015). In addition, based on a recent Chinese soil survey, the C:P ratio is generally less than 50 in the north of China (Tian et al., 2010), which is lower than the C:P ratio (usually 60) in microbial biomass. In addition, compared to natural systems, e.g., the forest and grassland, in the same region, the MBP in the intensive agricultural system is usually lower (Pal et al., 2013), which is related to the lower C:P ratio in the agricultural system. The intensive agricultural systems differs from the natural systems by having a smaller C input as the aboveground biomass is usually removed and the C source is mainly from root deposition and their residues which limit the C input (Oberholzer et al., 2014). This suggests there is an opportunity to apply some ecological concepts to the cropping system by enhancing the C input and closing the organic matter cycle (Bender et al., 2016).

## Managing soil C:P ratio to increase P microbial immobilization

Previous studies have demonstrated that adding C compounds to the soil can increase P microbial immobilization. Under soil conditions, adding glucose, plant residue, etc. increase MBP significantly in a short time (Kouno et al., 2002). In long term experiments, organic amendments also increase soil MBP (Liu et al., 2009). Considering the potential to improve MBP in the intensive agricultural system by increasing C:P ratio, here, an approach to reduce P loss from soil by enhancing microbial P immobilization through the addition of C-rich substrates is proposed. Various measures can be taken to increase the C input (Figure 1C): (1) application of organic fertilizer such as animal manure, which is the most popular organic fertilizer in China (Bai et al., 2016). As the poultry and livestock breeding industry has developed the amount of manure has greatly increased, China annually produces 3.8 billion tons of manure. This manure not only contains nitrogen and P, but also contains organic C compounds. However, much of this manure goes to waste in land fill. Applying animal manure to agricultural soil not only increases soil C, but is also environmentally friendly. Compost is another popular organic fertilizer in Chinese systems. Crop straw and other organic wastes are fermented and humified to form the humic compounds, which are easily utilized by microbes, and applied to soils as compost (Fan et al., 2006). Applying the organic fertilizer instead of part of chemical fertilizer has a great potential to increase the soil C content. (2) Retention of crop residues, by returning the crop straw back to the field, has potential to maintain and enhance the soil C content. The composition of straw includes a range of mainly C-rich compounds, while this may not be a useful source of essential nutrients for the crop, the enhanced soil carbon content will have many benefits. Though the retention of crop residues in cropping system has increased in recent years, only 31.6% of farmers retain their residues and the majority of straw is burnt or removed (Li et al., 2007). The straw returning rate needs to be increased in future. (3) Cover crops: in the non-crop growing season in the crop fields and in the orchard gardens, the cover plants can be cultivated and then plowed into soil when they are mature. This biological C fixation is also a potential approach to increase the soil C content. (4) Application of industrial byproducts with high C content: for example, in the sugar refining and monosodium glutamate industries, the byproducts usually contain highly labile C sources such as sugars and glutamic acid, which commonly detected in root exudates (Bais et al., 2006). These compounds are easily available C substrates for microbes and are likely to be potent promoters of microbial immobilization of P. (5) Application of domestic wastes: kitchen wastes, sewage sludge, and other urban waste streams produced by households usually contain high amount of organic matter which can also be applied in agriculture after processing to remove any harmful elements.

Taking these measures may go some way to promoting a circular economy on wastes and reduce the amount of waste from agriculture, animal husbandry, industry, and domestic households going to landfill. However, it is important to study how effective these various sources of C are at priming P immobilization by microbes and what impacts they have on the availability of P and other essential nutrients for crop productivity.

## Conclusions

Extensive application of P fertilizer to pursue high yields has increased soil P content in various intensive agricultural systems (including cereals, vegetables, and fruit orchards). Over-application of P fertilizer causes various environmental problems and it is imperative to understand how best to immobilize P in the soil to avoid its loss to the wider environment. Using soil microbial immobilization is a potentially efficient way to do this, but is usually limited by the low soil C:P ratio. Several measures can be taken to increase the C input: (1) application of organic fertilizer; (2) retention of crop residues; (3) cover crops; (4) application of industrial byproducts with high C content; and (5) application of domestic wastes.

## Author contributions

LZ, XD, and YP wrote the manuscript. TG and GF helped with writing, editing and finalizing the manuscript.

### Conflict of interest statement

The authors declare that the research was conducted in the absence of any commercial or financial relationships that could be construed as a potential conflict of interest.
